# Multi-Model Fusion-Based Hierarchical Extraction for Chinese Epidemic Event

**DOI:** 10.1007/s41019-022-00203-6

**Published:** 2023-01-02

**Authors:** Zenghua Liao, Zongqiang Yang, Peixin Huang, Ning Pang, Xiang Zhao

**Affiliations:** Laboratory for Big Data and Decision, National University of Defense Technology, Changsha, China

**Keywords:** COVID-19, Event extraction, Hierarchical extraction, Multi-model fusion

## Abstract

In recent years, Coronavirus disease 2019 (COVID-19) has become a global epidemic, and some efforts have been devoted to tracking and controlling its spread. Extracting structured knowledge from involved epidemic case reports can inform the surveillance system, which is important for controlling the spread of outbreaks. Therefore, in this paper, we focus on the task of Chinese epidemic event extraction (EE), which is defined as the detection of epidemic-related events and corresponding arguments in the texts of epidemic case reports. To facilitate the research of this task, we first define the epidemic-related event types and argument roles. Then we manually annotate a Chinese COVID-19 epidemic dataset, named COVID-19 Case Report (CCR). We also propose a novel hierarchical EE architecture, named *m*ulti-model *f*usion-based *h*ierarchical *e*vent *e*xtraction (MFHEE). In MFHEE, we introduce a multi-model fusion strategy to tackle the issue of recognition bias of previous EE models. The experimental results on CCR dataset show that our method can effectively extract epidemic events and outperforms other baselines on this dataset. The comparative experiments results on other generic datasets show that our method has good scalability and portability. The ablation studies also show that the proposed hierarchical structure and multi-model fusion strategy contribute to the precision of our model.

**Supplementary Information:**

The online version contains supplementary material available at 10.1007/s41019-022-00203-6.

## Introduction

At present, COVID-19 become a serious epidemic globally, and as of March 2022, there have been over 600 million confirmed COVID-19 cases, resulting in more than 6 million deaths [2]. Some surveillance efforts are devoted to using informative epidemic case reports to track and control the spread of COVID-19. However, conventional epidemic data processing methods require labor-intensive manpower input to effectively learn from past epidemic outbreaks. Therefore, how to build an efficient machine model to process epidemic data remains a challenging problem for healthcare workers and researchers.

**Fig. 1:**
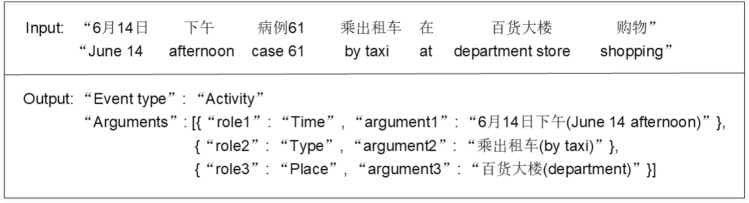
Example of EE

Recently, there has been extensive research on automatic epidemic EE. Epidemic event extraction (EE) aims to get structured epidemic event information, i.e., epidemic event trigger (with event type) as well as its corresponding arguments (with argument roles). It can be divided into two subtasks, i.e., event type identification (ETI) and event argument extraction (EAE). Figure 1 shows an example of epidemic EE. We input a piece of epidemic text into the model, and the output is the event type and the corresponding argument roles. Guo et al. [8] designed a three-stage pipeline method to extract epidemic events, achieving canto-level extraction. Mutuvi et al. [30] also propose a strong baseline and present a token-level dataset for multilingual epidemic EE.

**Fig. 2:**
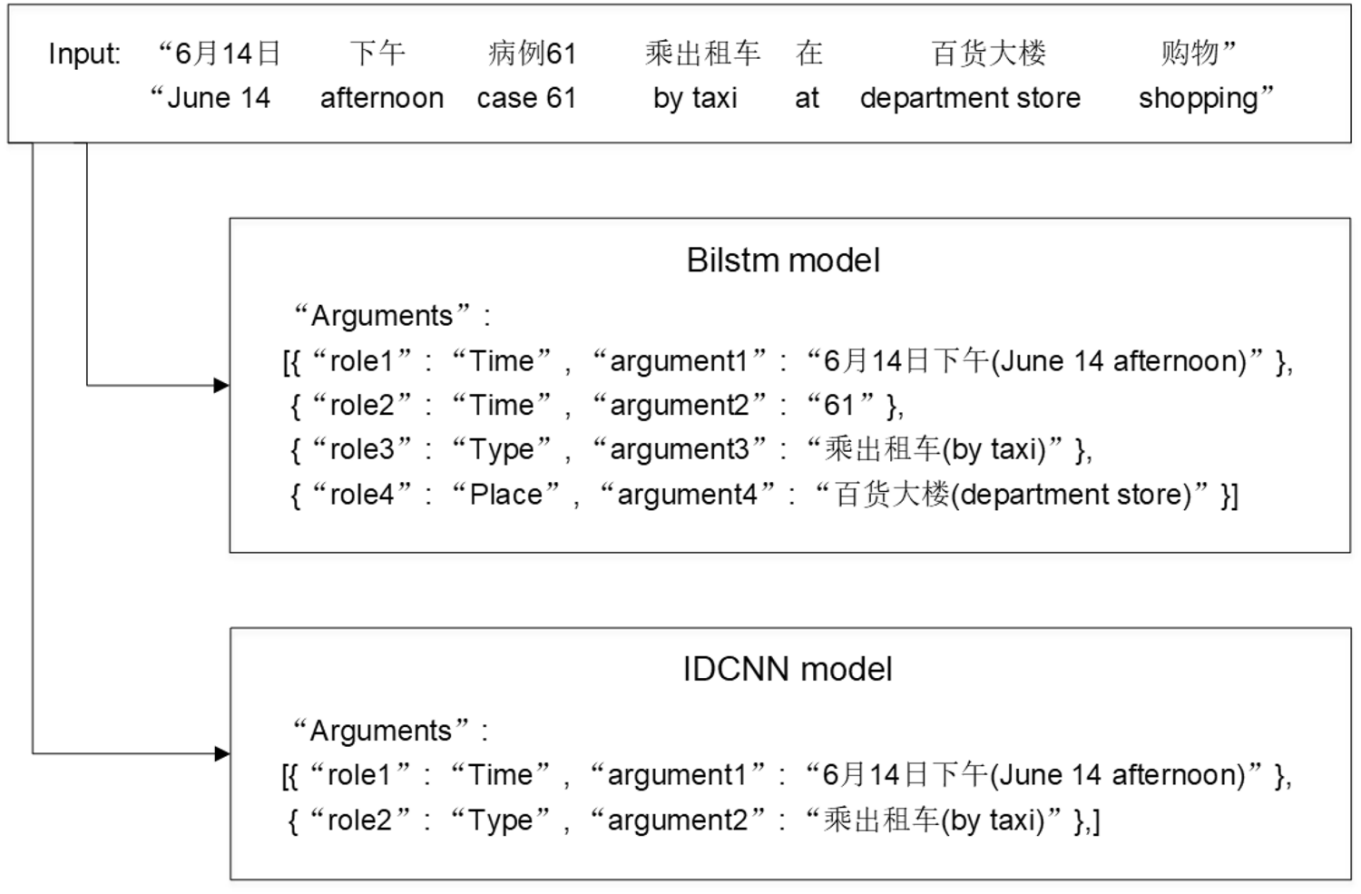
Extraction results of different EAE models

However, the previous models face the issue of recognition bias. Differing from the inductive bias in machine learning [6], we define recognition bias as the uneven recognition performance of different event types. Figure 2 shows the EAE results of the same sentence by different EAE models. It can be seen that the BiLSTM model [43] correctly identifies three arguments “June 14 afternoon”, “by taxi” and “department store”, but incorrectly identifies the Number “61” as Time. The IDCNN model [17] can correctly identify the two arguments “June 14 afternoon” and “by taxi”, but fails to recognize “department store”. There are several reasons for the phenomenon of error by BiLSTM and IDCNN: (1) The quality of the dataset is not high enough, and there are noise and conflicting data. (2) The parameter setting is not optimized enough, which leads to the defects of the model itself. (3) Different neural networks may do well in learning different perspectives from the events during the optimization process. Therefore, to solve this problem, we introduce a multi-model fusion strategy to solve this problem effectively. We retain the correct part of each model and filter out the wrong part, which will greatly improve the recognition accuracy.

In summary, existing epidemic-oriented methods have two problems: (1) There is no open-source dataset of manually annotated Chinese epidemic case reports. (2) The previous models have the problem of recognition bias, which leads to low recognition accuracy for specific event types. To facilitate the research on epidemic EE, we urgently need a large-scale, manually annotated dataset, and an efficient method to serve the domain of epidemic EE.

In this paper, we present COVID-19 Case Report (CCR), a large-scale manually annotated epidemic case report dataset that contains 25,870 events and 109,350 argument roles. We also propose a *m*ulti-model *f*usion-based *h*ierarchical *e*vent *e*xtraction (MFHEE) method for Chinese epidemic EE. MFHEE combines multiple advanced EE methods and retains the advantages of each model. MFHEE is a stronger epidemic EE method, which may further benefit other tasks related to COVID-19. We conduct thorough experiments on CCR under various settings and comprehensively evaluate the MFHEE method on different datasets, providing a promising research method for the study of epidemic EE.

This paper is organized as follows: after introduction in Sects. 1, 2 provide an overview of the work related to epidemic EE. In Sect. 3, we describe the dataset, and the architecture of the proposed method is elaborated in Sect. 4. In Sect. 5, we conduct ablation studies for hierarchical extraction and multi-model fusion, and compare our model with previous mainstream models in the field of epidemic EE. Finally in Sect. 6, we summarize some potential future work with our model.

## Related Works

Identifying and extracting text elements is one of the research hotspots in the domain of information extraction. In the past practice, most of the EE methods are based on dictionary [12], rule [13], or statistical machine learning [33]. However, these methods rely too much on human engineering, which limits their generalization. In recent years, with the maturity of deep learning research, deep neural network-based EE method has also become an important supporting technology for text element recognition [41]. Compared with traditional machine learning methods, the deep learning methods have the advantages of deeper network layers and more complex learning features. Besides, these methods have no further need manually construct features. The EE task is divided into ETI and EAE subtasks. ETI [8] classifies a piece of text into a target event type. EAE [35] aims to identify event arguments and classify their roles in the event.

There are two mainstreams of deep learning-based EE approaches: (1) Pipeline-based approach [3, 10, 21, 22, 24, 28, 39, 45] that first performs ETI and then identifies arguments base on the results of ETI. (2) Joint-based approach [16, 25, 29, 31, 44, 47, 49] that treats EE as a structure extraction task, and predicts event type and corresponding arguments at the same time. Although pipeline-based approaches are simpler and more flexible than joint-based approaches, they suffer from the problem of error propagation. Our multi-model fusion strategy can reduce the error at each stage, so we adopt the pipeline-based approach.

Many recent NLP systems use pre-trained language models as backbones, such as BERT [5] and ERNIE [36]. A variety of strategies for incorporating the language models output are used in EE systems. Some studies use the contextualized word embedding sequence as the input to a conditional random field entity extraction layer, while others add an entity-aware attention mechanism and pooled output states to a fully transformer-based model [27]. Here, we employ Google’s BERT language model [5] and Baidu’s ERNIE language model [36] to conduct experiments.

Recently, some research efforts are paid to explore the EE of COVID-19. Some long short-term memory networks-based methods [38] approach the task of epidemic EE from the perspective of classification of documents. Considering that COVID-19 case reports are usually presented in paragraphs and sentences, [8] realized the document-level epidemic EE. However, the majority of previous EE studies are usually based on generic datasets, and there are also limited COVID-19 datasets. These limitations affect the performance of epidemic EE models. Therefore, we introduce the CCR dataset to expand the corpus for the epidemic EE domain. To further improve the accuracy of epidemic EE, we also propose a Chinese EE method named MFHEE.

## CCR Dataset

In this section, we describe the process of constructing CCR in detail. The whole procedure can be divided into three steps: (1) We crawl a large number of unannotated Chinese epidemic case reports from websites, and define epidemic event types and argument roles according to the text characteristics. (2) We create a large candidate set via regularization matching and knowledge base alignment. (3) Human annotators filter out the wrong-labeled sentences and annotate the unlabeled events to finally obtain a clean CCR dataset.

### Event Definition

Event definition refers to the definition of event types and argument roles. According to the characteristics of epidemic case reports, we define five epidemic event types, including outbreak, case information, activity, confirmed, and affected. Taking activity as an example, its related arguments include time, place, type, and group. The definitions of all five event types are listed in Table 1.

**Table 1:** Event definition

Event type	Event argument
Outbreak	Time, City, New cases number, New suspected cases number
	Cumulative cases number, recovered cases number
Case information	Place, Name, Gender, Age, Origo, Occupation
	Address, Case, Relation
Activity	Time, Place, Type, Group
Confirmed	Time, Hospital
Affected	Time, Place, Type, Plate Number

- *Outbreak Event* An outbreak event is a specific occurrence related to the epidemic outbreak involving key elements. Its corresponding arguments include time, city, new cases number, new suspected cases number, cumulative cases number, and recovered cases number.
- *Case Information Event* A case information event is a specific occurrence related to the case information involving key elements. Its corresponding arguments include place (location of cases), name, gender, age, origo (native place of the case), occupation, address, case (other cases this case contact with), and relation (relationship between case and contacts).
- *Activity Event* An activity event is a specific occurrence related to the case activity involving key elements. Its corresponding arguments include time, place, type (mode of transportation of case), and group (companion of the case).
- *Confirmed Event* A confirmed event is a specific occurrence related to the cases of confirmed involving key elements. Its corresponding arguments include time and hospital (where the case was treated).
- *Affected Event* An affected event is a specific occurrence related to the epidemic spread involving key elements. Its corresponding arguments include time, place, type (type of vehicle involved in infection), and plate number.

### Candidate Set Construction

In the first step, we annotate the epidemic case reports preliminarily by regularization matching. For example, we annotate a piece of epidemic text as the “confirmed event” if it contains the word “confirm”. In addition, we annotate some structured event arguments via regularization matching, such as time, date, gender, and age.

Second, we harness Wikipedia as the external knowledge base (KB) to assist the entity annotations. Wikipedia is a large-scale KB, where a large proportion of entities are already linked to Wikipedia articles [46]. Besides, we employ the entity linking technique [42] to extract more entities in texts. Specifically, we adopt the named entity recognition HanLP [26] to find possible entity mentions, then match each mention with the name of an entity in KBs. All matched entities are annotated as event arguments. Finally, we obtain a candidate set containing 26,324 events and 32,422 event arguments.

### Human Annotation

Next, we invite well-educated annotators to filter candidate set data on the Label Studio platform which is a data annotation platform. The platform presents each annotator with one instance each time, by showing a piece of text and the preliminary annotations in the sentence. The annotators first judge whether the event type of the preliminary annotation is correct. Then determine if the argument roles are correct. If the annotation is incomplete, the annotator annotates all argument roles. Besides, the annotators mark an instance as negative if the sentence is incomplete.

Events are randomly assigned to an annotator, and each annotator consecutively annotates 20 instances of the same event type before switching to the next event type. To ensure annotation quality, each instance is annotated by at least two annotators. If two annotators have disagreements in this instance, it will be assigned to a third annotator. As a result, each instance has at least two same annotations, which will be the final decision [9]. After the annotation, we obtain the epidemic case report dataset CCR including well-annotated 25,870 epidemic event instances and 109,350 event arguments. Of these, 18,000 instances were used for training, 5870 for validation, and 2000 for testing.

### Data Analysis

In this section, we analyze various aspects of CCR to provide a deeper understanding of the dataset and the task of epidemic EE.

*Data Size* Table 2 shows statistics of CCR, including event types and event argument roles. We find that CCR is a large dataset in many aspects, including the number of sentences, event instances and arguments, especially in aspects of argument types. The CCR dataset contains 25,870 event instances, 109,350 event argument roles, and each event instance contains an average of 4.23 argument roles. We hope the large-scale CCR dataset could drive the development of the epidemic EE domain.

**Table 2:** Statistics of CCR

Event type	Outbreak	Case information	Activity	Confirmed	Affected
Amount	6326 (24.5%)	3624 (14.0%)	8917 (34.7%)	3580 (13.8%)	3423 (13.2%)
Event argument	Time	Place	Name	Type	.
Amount	32,427 (29.7%)	39,888 (36.5%)	16,322 (15.0%)	5224 (4.8%)	.

*Event Type* As shown in Table 2, CCR includes five event types in the epidemic field. A notable property of our dataset is that the events types cover a broad range of categories, including outbreak (24.5%), case information (14.0%), activity (34.7%), confirmed (13.8%), and affected (13.2%), which means that almost every epidemic event can be matched.

*Event Argument* As shown in Table 2, CCR covers a variety of event arguments, including time (29.7%), place (36.5%), name (15.0%), type (4.8%), etc. It also annotates different argument roles for each event type, enhancing the accuracy of event expression. Each event type has an average of 4.23 argument annotations.

## Method

In this section, we introduce a novel extraction method for epidemic events named MFHEE. MFHEE consists of three main parts: ETI, EAE, and Multi-Model Fusion.

1. ETI: we utilize three text classification models to detect epidemic event types. We select RNN-Attention, BERT-RCNN, and ERNIE-DPCNN as baselines of ETI.
2. EAE: we utilize three named entity recognition models to get the argument roles of the event. We select BERT-BiLSTM-CRF, IDCNN, and BERT-GlobalPointer as baselines of EAE.
3. Multi-model fusion: we utilize gradient-boosted decision tree (GBDT) [20] for multi-model fusion, which tackles the issue of recognition bias and improves the accuracy of the model.

**Fig. 3:**
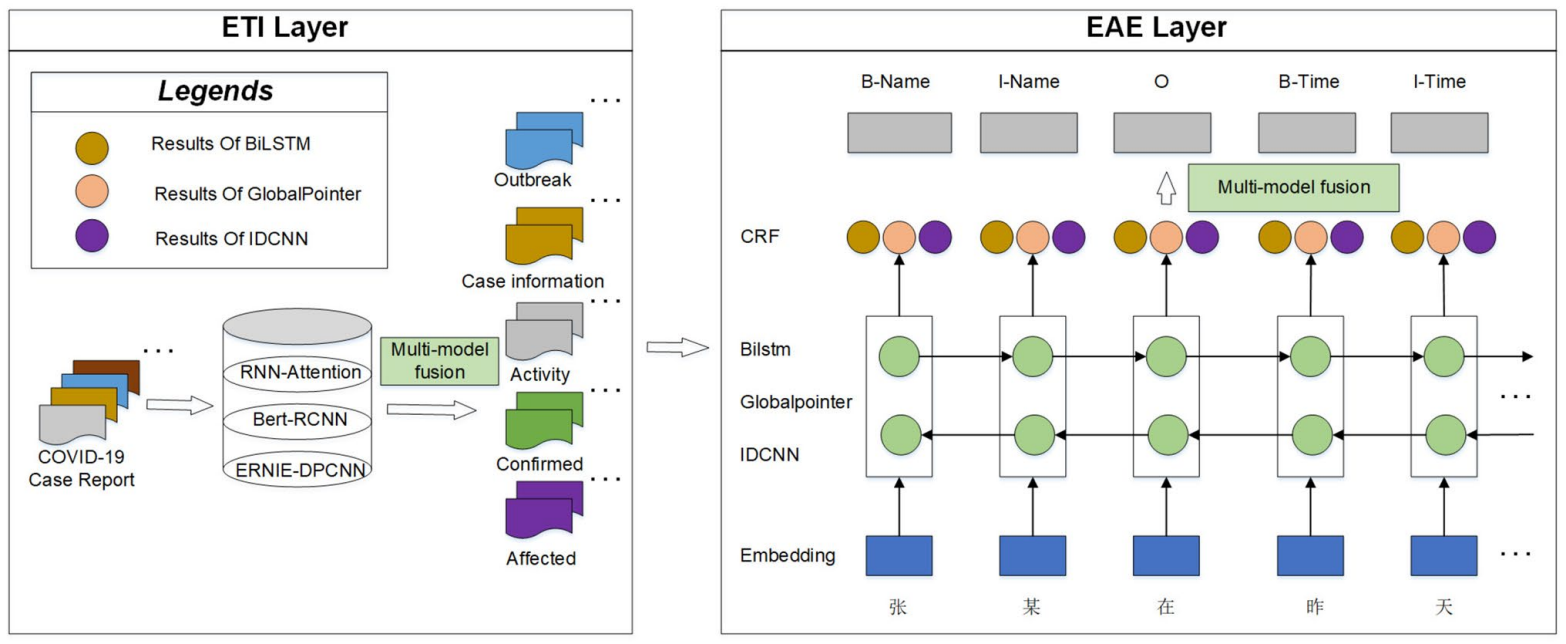
Overall architecture of MFHEE

The overall architecture of the MFHEE is illustrated in Fig. 3. Given a piece of epidemic text, ETI recognizes its event type. GBDT combines the results of the three models in ETI to obtain enhanced results. Then, EAE extracts the corresponding event argument roles according to the text event type. GBDT combines the results of the three models in EAE to obtain enhanced results.

### Baselines of ETI

*RNN-Attention* RNN-Attention first utilizes bidirectional LSTM to obtain long-distance advanced features. Then, the attention model is introduced to capture the internal dependence of sentences and calculate the contribution of different words to the text [23, 50]. Finally, the model outputs prediction results via softmax.

*BERT-RCNN* BERT is a transformer-based pre-trained language model which is widely applied to various NLP tasks [32]. RCNN [37] is a combination of recurrent and convolutional architectures. Two layers of LSTM [14] are employed in the architecture. One learns the context of words from left to right while the other learns contextually from right to left. The combination of BERT and RCNN can achieve better results.

*ERNIE-DPCNN* ERNIE utilizes multi-source data and prior knowledge for pre-training, which can capture the potential information in the training corpus more comprehensively [19]. DPCNN obtains more accurate local features of text through deep convolution, which can reduce the calculation and overcome the problem of difficulty in extracting long-distance text sequence dependencies [15].

**Fig. 4:**
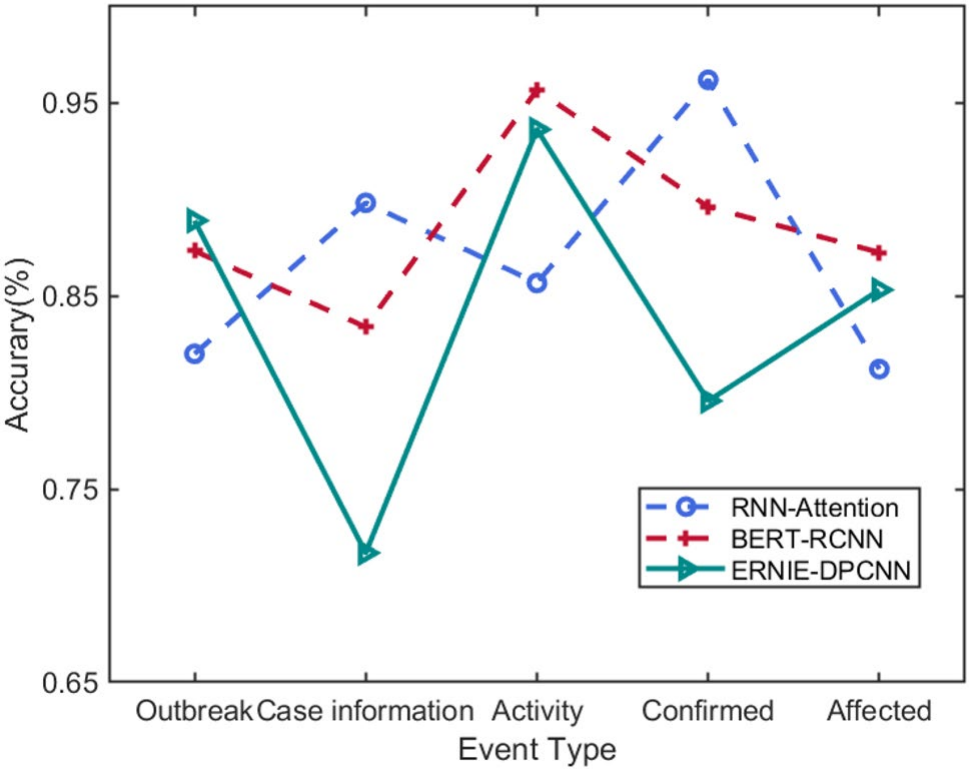
Results of ETI baselines

Figure 4 shows the accuracy of three baselines in extracting the types of different epidemic events. It can be seen that different models have uneven recognition performance in different event types. For example, RNN-Attention performs best on the confirmed event, but is weak in identifying the affected event. BERT-RCNN performs best on the activity event, but is weak in identifying the case information event. ERNIE-DPCNN performs best on the outbreak event, but is weak in identifying the confirmed event. We hope that the strengths and weaknesses of these baselines can complement each other. In Sect. 4.3, we adopt the method of multi-model fusion strategy to filter out the errors and get the correct results.

### Baselines of EAE

*BERT-BiLSTM-CRF* BERT-BiLSTM-CRF [7] utilizes the BERT to obtain the word vector corresponding to each input character in the corpus. Then the word vector sequence is input into a BiLSTM layer for semantic encoding, and finally, the output result is decoded through a CRF layer [43].

*IDCNN* IDCNN [40] improves the CNN structure by using holes. This method captures long-distance information of long text, which has better contextual and structured prediction capabilities than traditional CNNs.

*BERT-GlobalPointer* In pointer network designed for named entity recognition, we usually utilize two modules to identify the head and tail of the entity respectively, which leads to inconsistent training and prediction. GlobalPointer [21] treats both ends as a whole to deal with such inconsistencies. Therefore, GlobalPointer has a more global view.

**Fig. 5:**
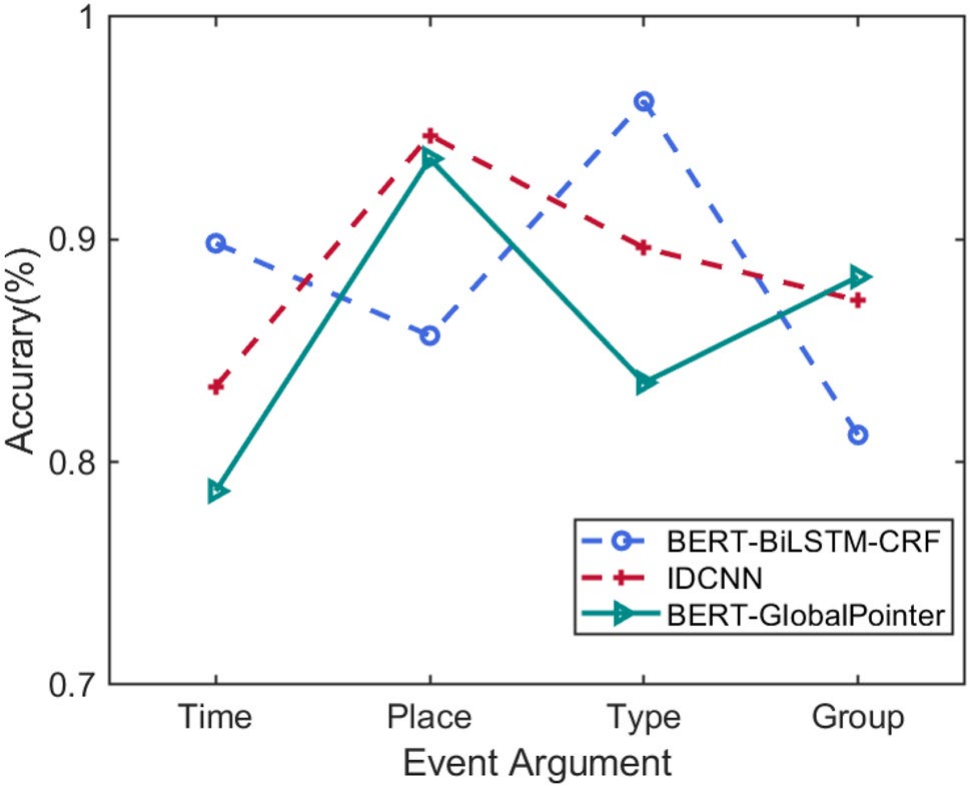
Results of EAE baselines

Figure 5 shows the accuracy of three baselines in extracting argument roles of activity event. It can be seen that different models have uneven recognition performance in different event argument roles. For example, BERT-BiLSTM-CRF performs best on the type argument but is weak in extracting group argument. IDCNN performs best on place argument but is weak in extracting time argument. BERT-GlobalPointer performs best on group argument but is weak in extracting time argument. Similar to ETI, we also adopt multi-model fusion strategy to solve the problem of recognition bias.

### Multi-model Fusion

We utilize GBDT algorithm to realize the multi-model fusion strategy. We harness the prediction results of baselines as the input to GBDT and GBDT outputs the final results via integrated learning. GBDT integrates the recognition advantages of baselines and solves the problem of recognition bias.

#### GBDT Algorithm

GBDT [20] is a machine learning algorithm using multiple decision trees (DTs) as base learners. A new DT increases the emphasis on the misclassified samples obtained from the previous DTs, and takes the residuals of the former DTs as the input of the next DT. Then, we harness the added DT to reduce the residuals so that the loss decreases following the negative gradient direction in each iteration. Finally, we determine the prediction result based on the sum of the results of all DTs. The result of the multi-model fusion is represented by *y*, and the result of each baseline is represented by *x*, where *N* is the number of samples of the training dataset. The goal of the decision tree is to solve the following formula:

1 $$\begin{aligned} F^* (x)={\text {argmin}} \sum _{\gamma }^{m} L\left( y_{i}, \gamma \right) , \end{aligned}$$

where $L\left( y_{i}, \gamma \right)$ is a loss function that reflects the accuracy of the training sample. $\gamma$ is the initial constant value and the DT model harness the addition function to predict the output:

2 $$\begin{aligned} F\left( x_{i}\right) =\hat{y}_{i}=\sum _{k} f_{k}\left( x_{i}\right) ;\quad f_{k} \in \phi , \end{aligned}$$

where $\phi \in \left\{ f(x)=w_{q(x)}\right\}$ is the space of classification regression tree, equivalent to the independent tree structure *q* and leaf weight *w* corresponding to each $f_k$.

Gradient propulsion method is adopted for parameter estimation to reduce residual error of the model:

3 $$\begin{aligned} \hat{y}=-\left\{ \frac{\partial L[y, F(x)]}{\partial F(x)}\right\} _{F(x)=F_{M-1}(x)}. \end{aligned}$$

The learning objective is defined as

4 $$\begin{aligned} L=\sum _{i} L\left( y_{i}, \hat{y}_{i}\right) +\sum _{k} \Omega \left( f_{k}\right) , \end{aligned}$$

where $\Omega \left( f_{k}\right) =\gamma J+\lambda w^{2} / 2$ is a classified regression tree function, and GBDT model is obtained through *M* iterations:

5 $$\begin{aligned} F_{M}(x)=\sum _{m=1}^{M} \quad \arg _{F(x)} \min \left[ \sum _{i} \quad L\left( y_{i}, \hat{y}_{i}\right) +\sum _{k} \quad \Omega \left( f_{k}\right) \right] . \end{aligned}$$

This loop is performed until the specified iterations times or the convergence conditions are met.

#### GBDT Validity Verification

**Fig. 6:**
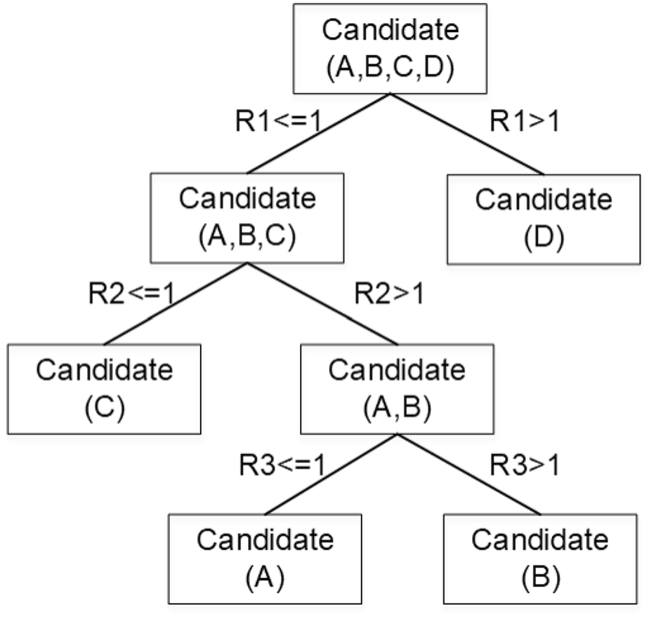
Example of DT structure. The set in parentheses is the candidate instance

To verify the validity of GBDT, we introduce a simple example. Table 3 shows the recognition results of four epidemic event instances by three ETI baselines. We construct a simple DT by taking the minimum squared error attribute value [1] as the splitting node, as shown in Fig. 6. It can be seen that the accuracy of results after the identification by GBDT is higher than that of all baselines, reaching 100%. In the actual process of constructing DT, we will construct multiple DTs and take the residual of the former DTs as the input of the next DT to achieve the minimum loss.

**Table 3:** GBDT recognition results, where “1” represents outbreak event, “2” represents activity event, and the underlined font represents recognition error

Instance	Correct	RNN-Attention	BERT-RCNN	ERNIE-DPCNN	GBDT
	Event type	Results (R1)	Results (R2)	Results (R3)	Results
A	1	1	2	1	1
B	2	1	2	2	2
C	1	1	1	2	1
D	1	2	1	1	1
Accuracy		50%	75%	75%	100%

## Experiment

To verify the effectiveness of MFHEE, we conduct ablation studies and comparative experiments to evaluate the MFHEE method on CCR dataset and generic datasets.

### Experiment Settings

*Model Hyper-parameters* The model configuration was selected using threefold cross-validation on the training set. Table 4 summarizes the selected configuration. Training loss was calculated by summing the cross entropy across all span and argument role classifiers. Models were implemented using the Python PyTorch module [27].

**Table 4:** Hyper-parameters for MFHEE model

Parameter	Value
Maximum sentence length	100
Batch size	128
Max epoch	100
Learning rate	0.001
Dropout rate	0.5
Optimizer	Adam
Segment embedding size	20
Char embedding size	100

*Evaluation Metrics* We conduct the experimental study based on two sets of evaluation metrics. The first set of metrics includes precision ratio (*P*), recall ratio (*R*), and F1-score (*F*1), which measures the performance of models. The other set of metrics includes false acceptance ratio ($\mathrm{FAR}$), false rejection ratio ($\mathrm{FRR}$), and detection cost function ($\mathrm{DCF}$), which measures the cost of recognizing errors in models [11].

6 $$\begin{aligned}&\mathrm{FAR}=\frac{\mathrm{FA}}{\mathrm{FA}+\mathrm{TR}}, \end{aligned}$$

7 $$\begin{aligned}&\mathrm{FRR}=\frac{\mathrm{FR}}{\mathrm{FR}+\mathrm{TA}}, \end{aligned}$$

8 $$\begin{aligned}&\mathrm{DCF}=C_{\text {miss}} \times P_{\text {miss}} \times P_{\text {target}}+C_{\mathrm{fa}} \times P_{\mathrm{fa}} \times \left( 1-P_{\text{ target }}\right) , \end{aligned}$$

where $\mathrm{TA}$ is the number of correct acceptances, $\mathrm{TR}$ is the number of correct rejections, *FA* is the number of false acceptances, and $\mathrm{FR}$ is the number of false rejections. $P_{\text{ miss }}$ is the loss rate, and $P_{\mathrm{fa}}$ is the false positive rate. $C_{\text {miss}}$ is the cost of a loss, and $C_{\mathrm{fa}}$ is the cost of a false positive, both of which are set to 1 in our experimental settings. $P_{\text{ target }}$ is the proportion of the error rejection rate and the error acceptance rate of prior knowledge. It is usually set as a constant value according to the specific application, which is set as 0.5.

### Ablation Studies

To verify the effectiveness of each module of MFHEE, we conduct two ablation studies. We mainly study the contribution of hierarchical extraction and multi-model fusion strategy to the model. Table 5 shows the two ablation models.

**Table 5:** Ablation models

Model	Interpretation
MFHEE	The full model
MFEE	Removing the hierarchical extraction
HEE	Removing the multi-model fusion strategy

1. MFHEE model is the full implementation of our model, which integrates hierarchical extraction and multi-model fusion strategy.
2. To verify the contribution of hierarchical extraction to our model, the multi-model fusion-based event extraction (MFEE) model removes the hierarchical extraction structure, which utilizes the baselines of EAE to extract event argument roles directly.
3. In order to verify the contribution of the multi-model fusion strategy, the hierarchical event extraction (HEE) model removes the part of multi-model fusion, and utilizes the highest F1-scores for ETI and EAE among all baselines for evaluation.

**Table 6:** Ablation studies results (%)

Model		*P*	*R*	F1-Score	FAR	FRR	DCF
ETI	HEE	90.65	89.32	89.98	12.93	15.99	18.34
	MFHEE	93.66	94.82	**94.24**	5.23	2.80	**7.89**
EAE	HEE	92.77	93.32	92.54	9.66	10.48	12.32
	MFEE	78.39	76.90	77.64	24.32	29.63	36.22
	MFHEE	95.36	94.99	**95.17**	3.33	1.63	**6.34**

Table 6 summarizes the results of ablation studies, where the values in bold refer to the best results for the indicators F1-Score and DCF on ETI and EAE tasks. In ETI and EAE tasks, the F1-Score of MFHEE is significantly higher than that of ablation models, and the error detection cost is significantly lower than that of ablation models. It can be seen from the experiments that both hierarchical extraction and multi-model fusion contribute greatly to the improvement in model accuracy. In addition, the two parts are interrelated, and removing either of them has a negative impact on the model.

### Comparative Experiments

To verify the advancement and scalability of MFHEE, we conduct comparative experiments to evaluate the MFHEE method on CCR dataset and generic datasets.

*Datasets* We select the following datasets for comparative experiments: (1) CCR is the epidemic EE domain dataset, which contains a wide variety of epidemic event types and argument roles; (2) DuEE1.0 [18] is the largest Chinese EE generic dataset; (3) CEC [48] is specially designed for Chinese EE. It is a small dataset, covering only five emergency event types.

*Contrasted Models* To verify the advancement and scalability of MFHEE, we set up the following models for comparison: (1) the three-stage pipeline EE method [8] realized the epidemic EE at the document-level; (2) DBRNN [34] extracts event triggers and arguments by dependency-bridge RNN; (3) BERT-DGCNN [4] is a BERT-based pipeline Chinese EE model.

**Table 7:** Comparative experiments results (%)

	Model		*P*	*R*	F1-Score	FAR	FRR	DCF
CCR	ETI	Three-stage (2022)	78.34	83.82	80.99	5.66	2.33	**7.86**
		MFHEE	93.66	94.82	**94.24**	5.23	2.80	7.89
	EAE	Three-stage (2022)	93.93	94.59	94.26	4.32	3.45	7.34
		MFHEE	95.36	94.99	**95.17**	3.33	1.63	**6.34**
DuEE 1.0	ETI	DBRNN (2018)	84.30	87.60	85.92	6.34	4.68	8.22
		BERT-DGCNN (2021)	87.30	86.32	**86.81**	5.20	5.11	7.98
		MFHEE	85.89	86.93	86.41	5.34	4.12	**7.31**
	EAE	DBRNN (2018)	78.10	84.70	81.27	7.84	5.22	9.61
		BERT-DGCNN (2021)	83.69	87.27	**85.44**	5.74	4.98	8.12
		MFHEE	81.66	88.95	85.15	5.46	2.19	**6.75**
CEC	ETI	DBRNN (2018)	89.20	82.70	85.83	6.32	3.23	**6.72**
		BERT-DGCNN (2021)	88.73	89.26	88.99	6.16	3.24	8.16
		MFHEE	87.97	90.26	**89.10**	5.56	3.47	6.98
	EAE	DBRNN (2018)	84.80	80.20	82.44	6.95	5.23	8.12
		BERT-DGCNN (2021)	88.21	87.32	87.76	5.98	2.94	7.96
		MFHEE	88.44	90.48	**89.45**	4.20	1.98	**6.45**

**Fig. 7:**
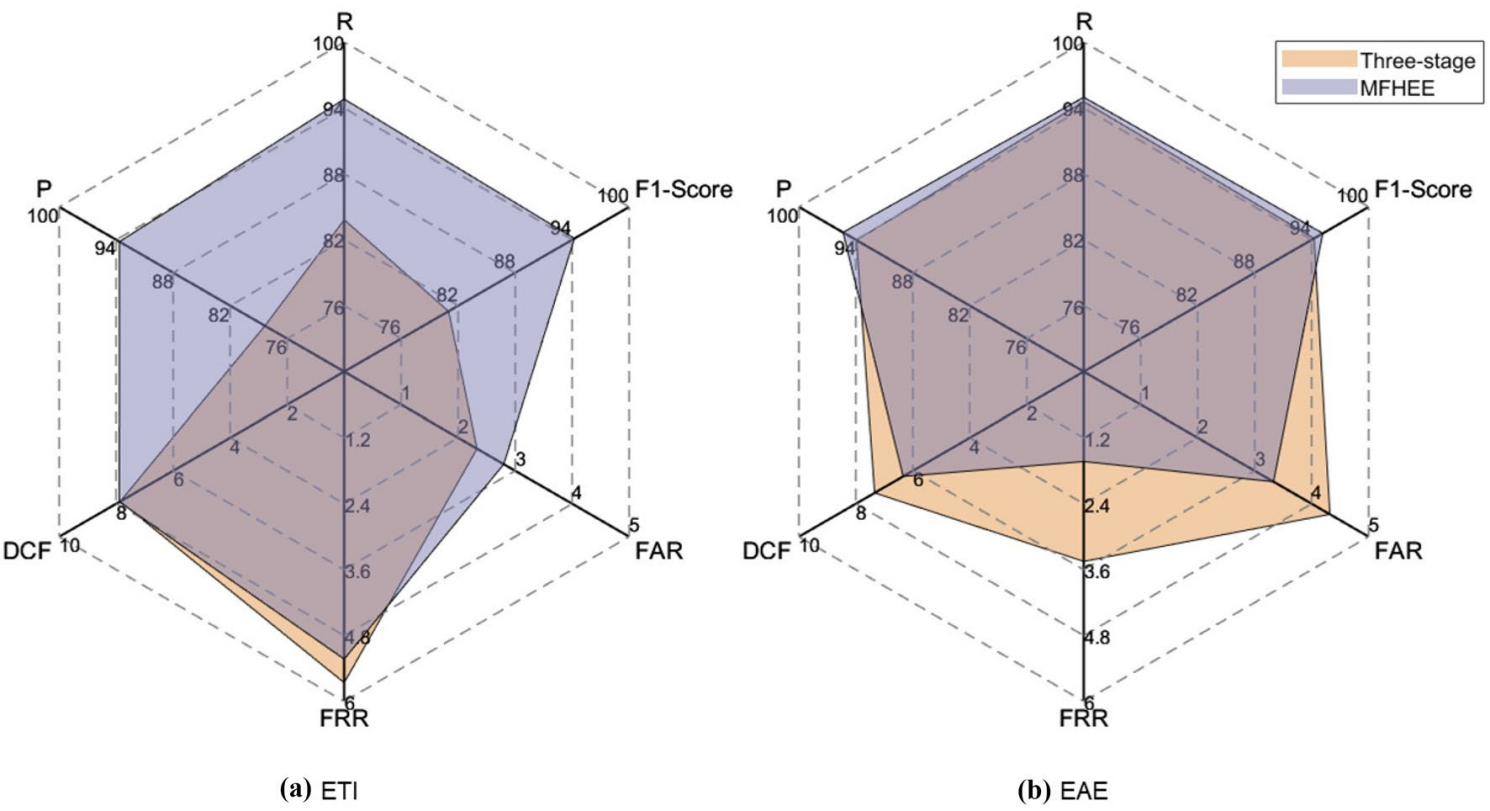
Radar diagram of comparative experiments results on CCR dataset (%)

*Overall Results* Table 7 shows the overall comparative experimental results on different datasets, where the values in bold refer to the best results for the indicators F1-Score and DCF on ETI and EAE tasks. Figure 7 shows the comparative experimental results of the MFHEE method on CCR dataset. In both the ETI task and ERE task, MFHEE has achieved the best results compared methods. However, the DCF of MFHEE method is slightly higher than three-stage method in the ETI task. The reason is that the three-stage method identifies event types at the document-level while MFHEE identifies event types at the sentence-level. Sentence has fewer features than document, making it harder to identify event types.

On the generic dataset DuEE 1.0, the MFHEE method achieves similar performance to the advanced models. On the small-scale dataset CEC, the MFHEE method is better than all compared models. It illustrates that our method can obtain better prediction results through the multi-model fusion strategy and hierarchical extraction. Moreover, our method has good scalability and portability in other domains.

## Conclusion

In this paper, we propose a new large and high-quality dataset CCR. This dataset provides a new point of view for the epidemic EE task. We also propose the MFHEE method to improve the accuracy of the epidemic EE model. This method solves the issue of recognition bias of previous EE models. The ablation studies suggest that both hierarchical extraction and multi-model fusion contribute greatly to our model. The comparative experiments suggest that the MFHEE method performs better than other EE baselines on CCR dataset and performs comparably to other advanced EE baselines on general datasets. Thus, we can use MFHEE as a stronger baseline for epidemic EE.

This paper leads to a variety of interesting future work, we are studying the effects of sentence segmentation on model accuracy. Besides, we are considering using transfer learning methods to simplify our model.

## Supplementary Information

Below is the link to the electronic supplementary material.

Supplementary file 1 (zip 67849 KB)

## Data Availability

We make CCR and the code for our baselines publicly available at https://github.com/liaozenghua/CCR-MFHEE.

## Abbreviations

EE: Event extraction
CCR: COVID-19 case report
MFHEE: Multi-model fusion-based hierarchical event extraction
ETI: Event type identification
EAE: Event argument extraction
ML: Machine learning
LSTMs: Long short-term memory networks
KB: Knowledge base
AM: Attention model
DTs: Decision trees
MFEE: Multi-model fusion-based event extraction
HEE: Hierarchical event extraction
GBDT: Gradient-boosted decision tree
COVID-19: Coronavirus disease 2019
