# Supplementary figures and images for: Multi-Model Fusion-Based Hierarchical Extraction for Chinese Epidemic Event
Source: Data Sci Eng. 2023 Jan 2;8(1):73–83. doi: 10.1007/s41019-022-00203-6 (PMC9807097; doi:10.1007/s41019-022-00203-6)

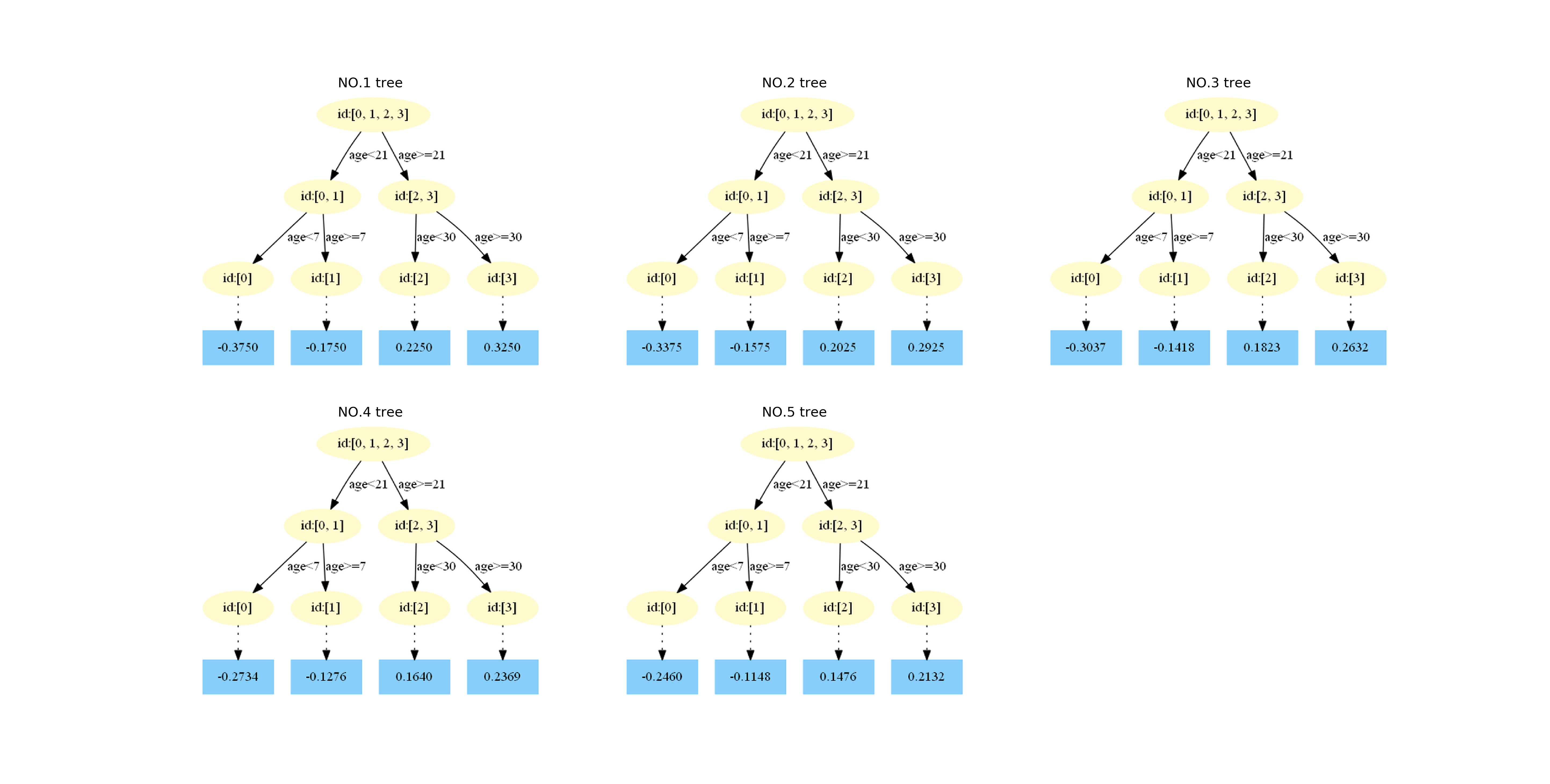

Supplement: Supplementary file 1 — (zip 67849 KB) [file 41019_2022_203_MOESM1_ESM.zip › gbdt_NER/展示图片/all_trees.png]
